# Evaluation of Intraspecies Interactions in Biofilm Formation by *Methylobacterium* Species Isolated from Pink-Pigmented Household Biofilms

**DOI:** 10.1264/jsme2.ME14038

**Published:** 2014-11-08

**Authors:** Fang-Fang Xu, Tomohiro Morohoshi, Wen-Zhao Wang, Yuka Yamaguchi, Yan Liang, Tsukasa Ikeda

**Affiliations:** 1 Department of Material and Environmental Chemistry, Graduate School of Engineering, Utsunomiya University, 7–1–2 Yoto, Utsunomiya, Tochigi 321–8585, Japan; 2 Laboratory for Food Safety and Environmental Technology, Institutes of Biomedicine and Biotechnology, Shenzhen Institutes of Advanced Technology, Chinese Academy of Sciences, Shenzhen 518055, China; 3 JST, CREST, 4–1–8 Honcho, Kawaguchi 332–0012, Japan

**Keywords:** *Methylobacterium*, household biofilm, pink biofilm, intraspecies interaction

## Abstract

Concern regarding household biofilms has grown due to their widespread existence and potential to threaten human health by serving as pathogen reservoirs. Previous studies identified *Methylobacterium* as one of the dominant genera found in household biofilms. In the present study, we examined the mechanisms underlying biofilm formation by using the bacterial consortium found in household pink slime. A clone library analysis revealed that *Methylobacterium* was the predominant genus in household pink slime. In addition, 16 out of 21 pink-pigmented bacterial isolates were assigned to the genus *Methylobacterium*. Although all of the *Methylobacterium* isolates formed low-level biofilms, the amount of the biofilms formed by *Methylobacterium* sp. P-1M and P-18S was significantly increased by co-culturing with other *Methylobacterium* strains that belonged to a specific phylogenetic group. The single-species biofilm was easily washed from the glass surface, whereas the dual-species biofilm strongly adhered after washing. A confocal laser scanning microscopy analysis showed that the dual-species biofilms were significantly thicker and tighter than the single-species biofilms.

Many environments within the common household provide conditions in which microorganisms can thrive, often resulting in the formation of biofilms. Bacteria have been cultured from many environments in and around homes, particularly from moist settings such as those around water pipes, showerheads, toothbrushes, spas, and bathrooms (4–6, 11, 14, 15, 19). Cases of opportunistic infections in humans have steadily increased over the past decade, and the source of infection often remains unidentified (2, 8, 10, 12). The increasing number of opportunistic infections is thought to correspond to the growing number of immunocompromised patients, many of whom self-medicate (8, 10, 18, 25). The formation of potential or adventitious pathogens in households poses a particular threat to such patients (9).

The formation of a pink-pigmented biofilm is often observed in wet areas within the household and may become a hygiene concern. Previous studies identified *Methylobacterium* as one of the dominant genera found in pink-pigmented household biofilms (11, 27, 32). Members of the *Methylobacterium* species are widely distributed and play important roles in both natural and man-made habitats, including plants, soils, air, dust, freshwater, aquatic sediments, marine environments, water supplies, and masonry (21, 31). *Methylobacterium* have been described as beneficial bacteria owing to their function in toxic pollutant biodegradation, the stimulation of germination, and plant development (16, 31); however, they are also known to be opportunistic human pathogens (12). *Methylobacterium* have been shown to exhibit higher stress-tolerance than other species found in biofilms, particularly under conditions of extreme nutrient limitation (32). Thus, it is considered laborious to remove already established pink-pigmented biofilms. Previous studies investigated the diversity of and interactions between household biofilms (6, 29, 32). However, bacterial interactions among the *Methylobacterium* species in household biofilms have not yet been elucidated in detail. In order to develop methods to control pink-pigmented household biofilms, it is considered essential to analyze these bacterial interactions and understand the mechanisms underlying biofilm development for the bacterial consortium of pink-pigmented biofilms. In the present study, we isolated and identified pink-pigmented bacteria from household biofilms, investigated the bacterial interactions enhancing biofilm formation, and examined the structures of both single and dual species biofilms.

## Materials and Methods

### Clone library analysis

Samples of visually confirmable pink slimes, which formed on a bathroom floor of a house, house K in Utsunomiya, Tochigi, Japan, were collected using a cotton swab at two different time points (July and December 2011). The head of the cotton swab with the pink slime was cut into pieces, and total DNA was extracted using a Cica Geneus DNA Extraction Reagent (Kanto Kagaku, Tokyo, Japan). The 16S rRNA genes from total DNA were amplified by PCR with GoTaq DNA polymerase (Promega, Madison, WI, USA) and the previously described primers, 63f (5′-CAG GCC TAA CAC ATG CAA GTC-3′) and 1387r (5′-GGG CGG TGT GTA CAA GGC-3′) (17). PCR was performed using the following cycling parameters: 94°C for 30 s, 50°C for 30 s, and 74°C for 1 min for 27 cycles. The PCR products were cloned into a pGEM-T easy vector (Promega). The 16S rRNA regions were sequenced using a BigDye Terminator ver. 3.1 and Applied Biosystems 3500 Series Genetic Analyzer (Applied Biosystems, Foster City, CA, USA). Closest type-strain 16S rRNA gene relatives to each clone sequence were determined using the RDP II sequence match tool (http://rdp.cme.msu.edu/seqmatch).

### Isolation and identification of pink-pigmented bacteria

Pink slimes were collected using cotton swabs from the bathrooms of five different houses, house M (collected in April 2011), house Y (May 2011), house T (July 2011), house S (August 2011), and house N (August 2011) in Utsunomiya, Tochigi, Japan (representative photos of the pink slimes are shown in Fig. S1). The isolation source of each isolate has been indicated with the last letter (M, Y, T, S or N) in the isolate name. The collected samples were serially diluted in sterilized water and spread onto MB medium (10 g L^−1^ peptone, 2 g L^−1^ yeast extract, 1 g L^−1^ MgSO_4_·7H_2_O, and 1% methanol) containing 1.5% (w/v) agar. After being incubated at 30°C for 1 week, the pink-pigmented colony that formed was collected and streaked onto new MB agar plates for single colony purification. To identify the bacterial species, the 16S rRNA gene fragments were amplified by PCR with GoTaq DNA polymerase and the primers 63f and 1387r. The resulting 16S rRNA sequences were aligned using the ClustalW program from DDBJ (30). A neighbor-joining tree was constructed using the NJplot software (23).

### Biofilm formation assay

Biofilm formation was determined using the previously described method with modifications (24). Bacterial strains were inoculated into the MB medium and incubated for 24 h at 30°C. Bacterial cells were collected by centrifugation and resuspended in fresh MB medium to an OD_600_ of 0.2. To form a mixed-biofilm, the cell suspensions of two different strains were mixed at a 1:1 OD_600_ ratio. Each cell suspension was diluted to an OD_600_ of 0.01 in fresh MB medium. Flat-bottom 96-well polystyrene microtiter plates (Thermo Fisher Scientific Inc. IL, USA) were used to examine biofilm formation. An aliquot of 100 μL of the diluted cell suspension was added to each well. After being incubated at 30°C for 48 h, 25 μL of a 0.1% crystal violet solution was added to each well. The plates were incubated at room temperature for 15 min and rinsed twice with sterile water. Crystal violet was dissolved in 100 μL of 99.5% ethanol, and biofilm formation was analyzed at 595 nm using a Spectra Max 250 spectrophotometer (Molecular Devices, CA, USA).

### Confocal laser scanning microscopy (CLSM) analysis

To prepare biofilm samples, bacterial strains were inoculated into the MB medium and incubated for 24 h at 30°C. Bacterial cells were collected by centrifugation and resuspended in fresh MB medium to an OD_600_ of 0.01. In order to form a mixed-biofilm, the prepared cell suspensions of two different strains were mixed at a 1:1 ratio. A volume of 3.5 mL of the cell suspensions was transferred to a 50-mL centrifuge tube. A sterile 18 × 18 mm glass cover slip was vertically dipped in the cell suspension and incubated statically for 3 d at 30°C to allow for biofilm formation. The biofilms that formed were stained using a FilmTracer LIVE/DEAD Biofilm Viability kit (Invitrogen, Carlsbad, CA, USA). Staining was conducted according to a standard protocol. Briefly, fluorescence-staining solutions were prepared by adding 3 μL of the SYTO9 stain for live organisms and 3 μL of the propidium iodide stain for dead organisms into 1 mL of sterilized water. A volume of 200 μL of the staining solution was then added on top of the biofilm, which was then incubated in the dark for 25 min. Samples were rinsed with sterilized water and examined using a FluoView FV10 laser scanning confocal microscope (Olympus, Tokyo, Japan). Simulated fluorescence projection images were generated using the FluoView application software package (Olympus). *Nucleotide sequence accession numbers* The nucleotide sequences reported in this study were deposited in the DDBJ, ENA, GenBank databases with the following accession numbers: AB900964–AB900979 (for *Methylobacterium* isolates), AB979860–AB979864 (for other pink-pigmented isolates) and LC002341–LC002519 (for clones).

## Results and Discussion

### Phylogenetic analysis of clone libraries

To determine the composition of the bacterial community in the pink slime, clone libraries of the 16S rRNA genes were prepared and sequenced from the pink slime that formed on the bathroom floor. A total of 87 and 92 clones were sequenced from the pink slime samples collected from the same position of house K in July and December 2011, respectively. We took relatedness clusters with 97% or higher sequence identity to correspond to a species-level relationship and clusters with 95% or higher sequence identity to correspond to a genus-level relationship (11). *Methylobacterium* and *Sphingomonas* were the predominant genera found in the pink slime at the two evaluated time points. Regarding the genus *Methylobacterium*, clone sequences that showed similarity to *M. brachiatum* B0021^T^ (accession no. AB175649) were mainly found in the clone libraries of July and December 2011 (*n* = 20 and 47, respectively). The same number (*n* = 3) of clone sequences that showed similarity to other *Methylobacterium* species were also found in both clone libraries. Regarding the genus *Sphingomonas*, clone sequences that showed similarity to *S. aquatilis* JSS-7^T^ (accession no. AF131295) were mainly found in the clone libraries (*n* = 9 and 15, respectively). Clone sequences that showed similarity to other *Sphingomonas* species were also found in both clone libraries (*n* = 7 and 4, respectively). The sequences of the other clones (*n* = 48 and 23, respectively) showed low identities (<95%) with the 16S rRNA sequences of the type strains. These 48 or 23 clones were divided into 10 and 14 groups having sequence identities above 97%, respectively. These results demonstrated that the pink slime that formed on the bathroom floor consisted of many kinds of bacteria, which primarily belonged to the genera of *Methylobacterium* or *Sphingomonas*. The genera of *Methylobacterium* and *Sphingomonas* were previously detected as the dominant members in biofilms that formed on vinyl shower curtains (11). This previous study also reported the co-existence of two or three species of *Methylobacterium* in the biofilms as shown here. However, *M. brachiatum*, which was mainly found in this study, was not detected in the previous study.

### Isolation and identification of pink-pigmented bacteria

**Fig. 1:**
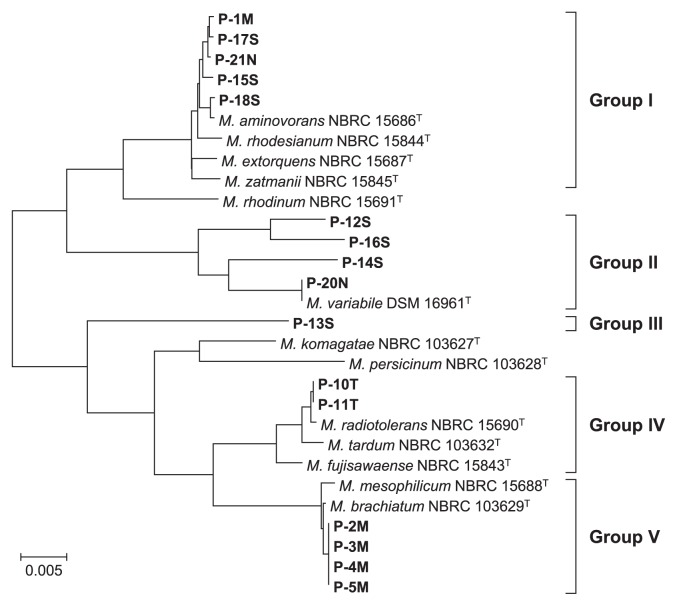
Neighbor-joining trees of 16S rRNA gene sequences obtained for *Methylobacterium* strains. Thirteen type strains of the genus *Methylobacterium* and 16 isolates were used for the phylogenetic analysis with the 16S rRNA gene. The bacterial isolates used in this study are shown in bold. The scale bar represents 0.005 substitutions per nucleotide position. Bacteria were divided into 5 groups based on phylogenetic relationships.

To analyze the cultivable bacterial composition of pink slime, samples of pink-pigmented bacteria were taken from the bathrooms of five different houses and screened. 16S rRNA gene fragments were amplified and sequenced to identify the pink-pigmented bacterial isolates. A total of 16 out of the 21 pink-pigmented bacterial isolates were assigned to the genus *Methylobacterium*. Previous studies identified the genus *Methylobacterium* as a major isolate in household biofilms. Our results confirmed that the genus *Methylobacterium* was one of the dominant groups of pink-pigmented bacteria. A BLAST search revealed that the 16S rRNA sequences of the other less-represented isolates (1 strain each) showed identities to that of *Pedobacter suwonensis* 15–52^T^ (95.4%), *Caulobacter henricii* ATCC 15253^T^ (79.7%), *Terriglobus roseus* KBS 63^T^ (88.3%), *Roseomonas rosea* 173–96^T^ (92.7%), and *Hymenobacter rigui* WPCB131^T^ (95.6%), respectively. These bacterial species have been previously identified as pink-pigmented bacteria (1, 3, 7, 13, 26). Based on the results of the phylogenetic analysis with the 16S rRNA genes, we divided the sixteen *Methylobacterium* strains into five groups (Fig. 1). Group I showed over 96% identity to *M. zatmanii* NBRC 15845^T^, *M. extorquens* NBRC 15687^T^, *M. rhodesianum* NBRC 15844^T^, and *M. aminovorans* NBRC 15686^T^. Although one group II strain, P-20N, showed 100% identity to *M. variabile* DSM 16961^T^, other group II and III strains did not show similarity to the type strains belonging to the genus *Methylobacterium*. Group IV showed 98.8% identity to *M. radiotolerans* NBRC 15690^T^ and group V showed 99.5% identity to *M. brachiatum* NBRC 103629^T^ and *M. mesophilicum* NBRC 15688^T^.

### Intraspecies interactions contributed to the formation of robust biofilms

**Fig. 2:**
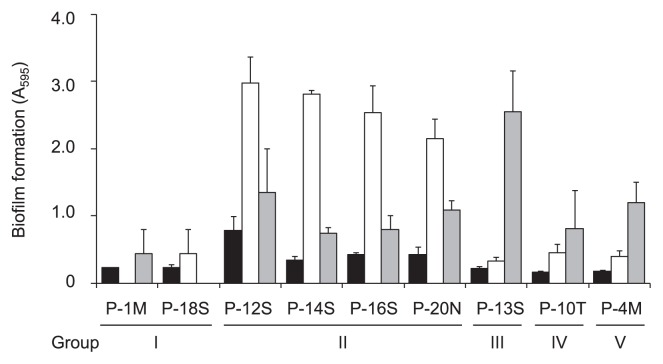
Biofilm formation by *Methylobacterium* strains on 96-well polystyrene plates. The overnight cultures of *Methylobacterium* strains were diluted and transferred to each well of a 96-well polypropylene microtiter dish. After being incubated at 30°C for 48 h, biofilms were stained with crystal violet and estimated by analyzing absorbance at 595 nm. Six wells of each sample were used to measure biofilm formation, and error bars indicate standard deviations. Single biofilms (black bars), mixed biofilms with P-1M (white bars), and mixed biofilms with P-18S (grey bars) are shown.

The formation of biofilms from the pink-pigmented bacterial isolates was evaluated on a polystyrene plastic surface. In the biofilm formation assay, *Methylobacterium* sp. P-1M and P-18S from group I, P-12S, P-14S, P-16S, and P-20N from group II, P-13S from group III, P-10T from group IV, and P-4M from group V, were selected. The selected strains were inoculated into MB medium in 96-well polystyrene plates and incubated for 48 h. The results of the biofilm assay revealed that all of the tested strains formed low-level biofilms (Fig. 2). Previous studies showed that mixed-species biofilms with interspecies interactions were often thicker and more physically and physiologically robust than single-species biofilms (27, 33). Therefore, we examined dual-species biofilm formation by co-incubating the selected *Methylobacterium* isolates as above along with the other pink-pigmented isolates, *P. suwonensis*, *C. henricii*, *T. roseus*, *R. rosea*, and *H. rigui*. However, none of the combinations demonstrated enhanced biofilm formation (data not shown). We then examined dual-species biofilm formation by mixing two of the nine selected *Methylobacterium* isolates with all possible combinations. The amount of the biofilms formed by the group I strain, P-1M, significantly increased when it was co-cultured with the four group II strains (Fig. 2). In addition, biofilm formation by another group I strain, P-18S, was also enhanced by co-culturing with a group III strain, P-13S (Fig. 2). However, mixed biofilm formation by two group I strains, P-1M and P-18S, was not enhanced over that by a single strain (Fig. 2). Furthermore, any other combination of the selected nine *Methylobacterium* isolates did not enhance biofilm formation (data not shown). Interspecies interactions within biofilm communities have been widely reported in previous studies (22, 27, 28, 33). To the best of our knowledge, this is the first study to demonstrate that intraspecies interactions in the genus *Methylobacterium* enhanced biofilm formation.

**Fig. 3:**
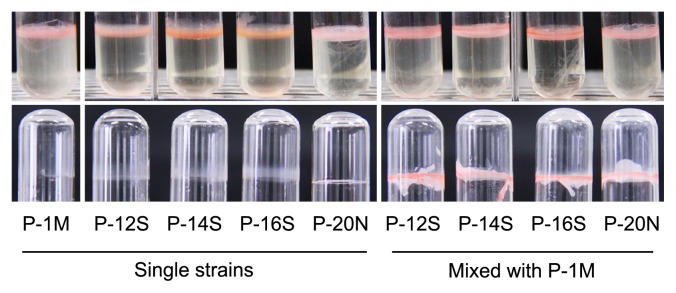
Single- and dual-species biofilms formed on the surface of glass tubes. *Methylobacterium* strains P-12S, P-14S, P-16S, and P-20N were inoculated with or without P-1M in 4 mL of MB medium in glass test tubes and statically incubated for 3 d at 30°C. The pellicle-like biofilms formed by the single- or dual-species are shown in the upper part. The remaining biofilms after washing are shown in the lower part.

To investigate the phenotypes of enhanced biofilm formation, P-1M, P-12S, P-14S, P-16S, and P-20N were inoculated either solely or dually in 4 mL of MB medium in glass test tubes. After 3 d of static cultivation, the formation of a pellicle-like biofilm was observed at the liquid-gas interface of both the single- and dual-species cultures (Fig. 3). When the medium was removed and the biofilms were washed twice with the sterilized water, the single-species biofilm was easily washed from the glass surface, whereas the dual-species biofilm adhered strongly after washing (Fig. 3). These results showed that intraspecies interactions played an important role in the formation of the robust biofilms by the *Methylobacterium* strains.

### CLSM analysis showed structural differences between single- and dual-species biofilms

**Fig. 4:**
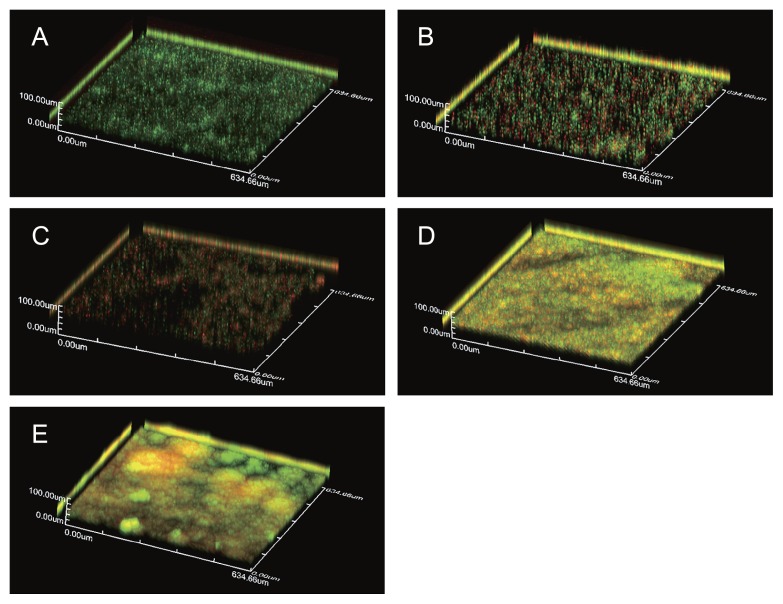
CLSM analysis of a single P-1M biofilm (A), single P-14S biofilm (B), single P-20N biofilm (C), mixed P-1M and P-14S biofilm (D), and mixed P-1M and P-20N biofilm (E). Single- and dual-biofilms were formed on sterile glass cover slips and stained using a FilmTracer LIVE/DEAD Biofilm Viability kit. Samples were rinsed with sterilized water and examined using a FluoView FV10 laser scanning confocal microscope. Live cells are stained green and dead cells are stained red. The z-axis scale bar represents 100 μm.

To examine the three-dimensional structures of single- and dual-species biofilms, we selected the three *Methylobacterium* strains, P-1M, P-14S, and P-20N. A LIVE/DEAD biofilm viability kit was employed to assay bacterial viability in the biofilms. The single-species biofilms of P-1M, P-14S, and P-20N were comparatively scattered with low cell density (Fig. 4A, B, C). However, the dual-species biofilms of P-1M with P-14S or P-20N were significantly tighter with a high cell density (Fig. 4D, E). Furthermore, several mushroom-shaped structures were observed in the dual-species biofilms of P-1M and P-20N (Fig. 4E). These results showed that intraspecies interactions affected the robustness of the biofilms and structure formation in *Methylobacterium* strains. Large amounts of dead cells were observed in the single species biofilm formed by P-14S or P-20N (Fig. 4B, C). It has been widely reported that extracellular DNA (eDNA), which is released from dead cells, plays an important role in biofilm formation (20). eDNA adsorbs to the cell surface, promoting adhesion to abiotic surfaces. In order to form a robust biofilm, a proper ratio of live/dead cells may be necessary to maintain a suitable amount of eDNA. Therefore, we assumed that the dead cells of P-14S and P-20N played the role of a supplier of eDNA in their mixed species biofilm.

## Conclusion

Our results revealed that the genus *Methylobacterium* is one of the dominant bacteria found in the pink-pigmented slime that widely forms in bathrooms. Although most *Methylobacterium* strains independently formed poor biofilms, some specific *Methylobacterium* strains interacted with other phylogenetically different *Methylobacterium* strains to enhance biofilm formation. The results of the clone library analysis showed that *M. brachiatum* and the other *Methylobacterium* species co-existed in the same pink slime. It is highly possibility that intraspecies interactions between different *Methylobacterium* species contributed to the formation of the strong biofilms found in pink-pigmented household biofilms. Future studies to more clearly understand the molecular mechanisms underlying intraspecies interactions for the development of dual-species biofilms may provide useful information for the development of methods to control pink-pigmented household biofilms.

## Supplementary Information
