# Supplementary material for: Evaluation of Intraspecies Interactions in Biofilm Formation by Methylobacterium Species Isolated from Pink-Pigmented Household Biofilms
Source: Microbes Environ. 2014 Nov 8;29(4):388–92. doi: 10.1264/jsme2.ME14038 (PMC4262362; doi:10.1264/jsme2.ME14038)

Supplemental figure legends

Fig. S1. Pink slimes collected from bathrooms in houses M and N. Black arrows indicate the formation of pink slimes.

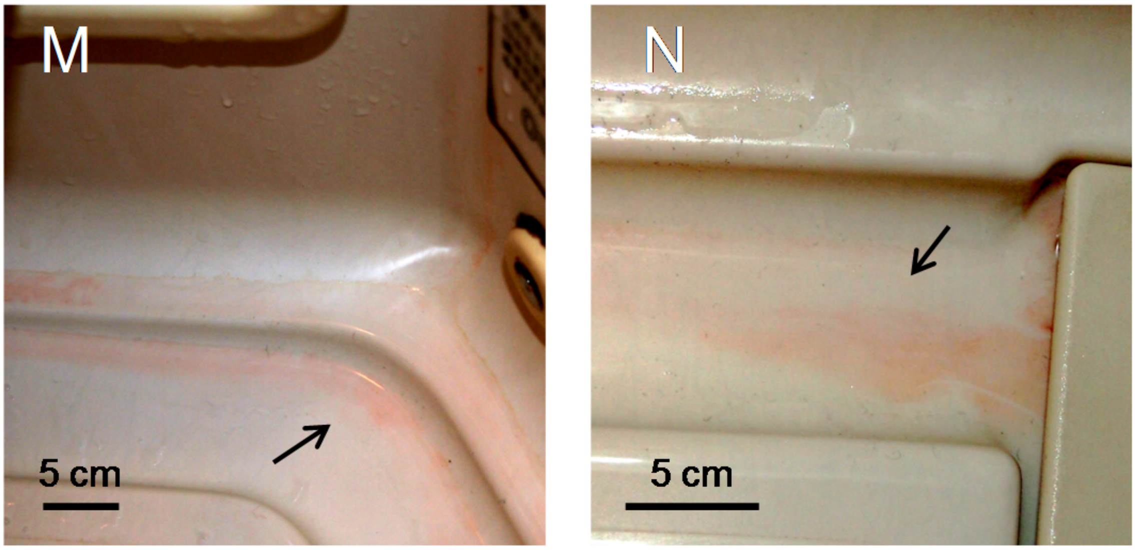

Supplement: Supplementary file 1 [file 29_388_s1.pdf]
